# Influence of cognitive abilities on literacy skills in a Korean–Japanese bilingual child with developmental dyslexia

**DOI:** 10.3389/fpsyg.2022.942775

**Published:** 2022-11-04

**Authors:** Ami Sambai, Yeongsil Ju, Akira Uno

**Affiliations:** ^1^Faculty of Human Sciences, University of Tsukuba, Ibaraki, Japan; ^2^LD/Dyslexia Centre, Chiba, Japan

**Keywords:** bilingual, developmental dyslexia, visual skills, Korean, Japanese

## Abstract

Some individuals with developmental dyslexia show dissociation in reading skills between languages. The occurrence of dissociation depends on differences in the orthographic characteristics and cognitive demands of languages. This article reports on a Korean–Japanese bilingual and biliterate boy, SJ, with developmental dyslexia (aged 11 years), who displayed dissociation between Korean and Japanese in reading and writing accuracy. This study aimed to discuss possible accounts for the profile of his literacy skills from orthographic and cognitive perspectives. To accomplish this aim, we measured SJ’s literacy skills, receptive vocabulary, and cognitive abilities (i.e., phonological skills, naming speed, and visual skills) in both Korean and Japanese. Then, we compared his skills to those of monolingual and bilingual children. In terms of accuracy, his reading skills in Korean did not differ significantly from those of bilinguals, although they were lower than Korean monolinguals. His spelling skills were within the average range for Korean monolinguals and bilinguals. In contrast, his reading and writing accuracy levels in Japanese were low compared to both Japanese monolinguals and bilinguals. Moreover, his reading and writing deficits were more remarkable in Japanese kanji. However, his cognitive profile was similar between languages. Specifically, he showed deficits in phonological skills and naming speed in both languages, as well as deficits in visual skills. These results were explained by the facts that visual skills are one of the significant predictors of reading and writing accuracy in Japanese but not in Korean, and that visual skills are a key in learning kanji. Thus, our case, SJ, supports the cognitive account, namely, the idea that different cognitive demands on the development of literacy skills can cause dissociation in literacy skills (especially in terms of accuracy) in bilingual children.

## Introduction

There are several studies on the development of literacy skills in bilingual children and second-language (L2) literacy skills in biliterate children. Their primary focus is often on the development of L2 literacy skills in English or the differences in the development of reading skills among European languages. In comparison, there are fewer studies on bilingualism or biliteracy among Asian languages.

Some studies have focused on bilingual or biliterate children with developmental dyslexia. Developmental dyslexia is the most prevalent learning disability. Its characteristics include difficulties with accurate and/or fluent word recognition and poor spelling and decoding abilities (Lyon et al., 2003). It is globally accepted that a cause of developmental dyslexia is deficits in cognitive abilities, which are requisite for literacy acquisition. As the definition of developmental dyslexia (Lyon et al., 2003) states, an impairment in phonological skills (i.e., the ability to recognize, identify, or manipulate phonological structures within a word) is widely accepted as a cause of developmental dyslexia in English. This article reports on a Korean–Japanese bilingual boy, SJ (11 years old), with developmental dyslexia. He showed differences and similarities in his literacy profiles between Korean and Japanese. This study discusses possible accounts for his literacy skills from orthographic and cognitive perspectives.

### Variation of reading abilities between different languages

In cross-linguistic studies across European languages, the consistency of letter-to-sound correspondences influences the development of a child’s reading skills. Generally, the reading acquisition of monolingual children in inconsistent orthographies (e.g., English) takes more time and is more difficult than in consistent orthographies (e.g., Italian, German; e.g., Seymour et al., 2003). The prevalence of reading deficits in consistent orthographies is lower than in opaque ones (e.g., Landerl et al., 1997). In consistent orthographies, the main reading problem of monolingual children with developmental dyslexia lies in fluency rather than accuracy (Wimmer, 1993).

The consistency of letter-to-sound correspondences affects the reading development of bilingual children who use multiple European languages. According to Lallier et al. (2014), French-Spanish bilingual children with developmental dyslexia demonstrated deficits in reading accuracy, which were primarily visible in their inconsistent orthography (French) rather than their consistent one (Spanish).

Similarly, orthographic characteristics can influence the reading acquisition of monolingual and bilingual children in Asian countries. For example, some Chinese children with developmental dyslexia have reading deficits in both Chinese and English, while others show reading deficits in either Chinese or English (e.g., Ho and Fong, 2005; McBride-Chang et al., 2012; Li et al., 2018). In Japan, Tsutamori et al. (2012) reported that a Japanese monolingual child with developmental dyslexia showed reading deficits in English but not in Japanese. Further, Wydell and Butterworth (1999) reported on an English-Japanese bilingual boy with developmental dyslexia, AS. AS also showed reading deficits in English, but not in Japanese. Thus, some children with developmental dyslexia show dissociation of reading abilities between languages.

### Explanations for dissociation of reading abilities between different languages

The variation of reading abilities between different languages is explained from orthographic and cognitive perspectives. Generally, the consistency of letter-to-sound correspondences is considered an orthographic factor for the variation in reading abilities among European languages (e.g., Seymour et al., 2003; Ziegler and Goswami, 2005; Landerl et al., 2013). In addition, the size of the phonological unit in sounds corresponding to letters is also taken as an orthographic factor (Wydell and Butterworth, 1999).

Wydell and Butterworth (1999) proposed the hypothesis of granularity and transparency to explain the dissociation of reading abilities between languages. In their hypothesis, they use the term “transparency” for the consistency of letter-to-sound correspondences, and the term “granularity” for the phonological unit in sounds corresponding to letters. According to the hypothesis, the difficulty in the acquisition of letter-to-sound correspondences depends on the dimensions of transparency and granularity. As a result, the prevalence of developmental dyslexia varies with these dimensions. Specifically, in the transparency dimension, when the letter-to-sound correspondences are transparent (consistent), children will easily acquire the letter-to-sound correspondences. Consequently, transparent orthographies will not produce a high incidence of developmental dyslexia regardless of the granularity (e.g., phoneme, mora, syllable, etc.). In the granularity dimension, when the phonological unit in sounds corresponding to letters is large, children will easily acquire the letter-to-sound correspondences, regardless of the transparency. As a result, there will not be a high incidence of developmental dyslexia in any orthographies where the phonological unit is large. Thus, since orthographic characteristics influence the difficulty of reading acquisition, it is expected that a child will show reading deficits only in language, wherein reading acquisition is relatively difficult.

English is an inconsistent orthography, and its granularity is small (i.e., phonemes). Therefore, the hypothesis of granularity and transparency predicts that English will cause a high incidence of developmental dyslexia. However, the hypothesis predicts that both Japanese writing systems, Japanese kana and kanji, will not cause developmental dyslexia to that extent. This is because Japanese kana is a consistent orthography, and the phonological unit of Japanese kanji is large (see details in section Predictions from the orthographic perspective). In fact, the English-Japanese bilingual case of Wydell and Butterworth (1999), AS, showed developmental dyslexia in English alone. Therefore, AS’s literacy profile matched the prediction from the hypothesis of granularity and transparency.

However, there is an alternative account for AS’s dissociation of reading skills. From a cognitive perspective, the dissociation of reading abilities between languages can be explained by different cognitive demands between languages. The contribution of cognitive abilities to reading development differs across languages. For example, phonological skills are related to reading development in European languages and Chinese. However, the role of phonological skills in reading development in Chinese becomes more important with school years (Wei et al., 2014), while its role in European languages emerges in the early stage of reading development (Clayton et al., 2020). In addition, phonological skills at the phoneme level are necessary for reading acquisition in English (e.g., Stuart and Masterson, 1992; Share, 1995; Hulme et al., 2002), but not in Chinese (Ho and Fong, 2005). Similarly, the phonological unit in Japanese writing systems is mora, but not phoneme. Therefore, phonological skills at the mora level are necessary for reading acquisition in Japanese (e.g., Uno et al., 2009; Inomata et al., 2016; Hamada and Uno, 2021), while phonological skills at the phoneme level are not important.

The cognitive perspective predicts dissociation of reading abilities between different languages, as follows: If a cognitive ability contributes to reading development in a language (Language A), but not another language (Language B), a child with impairment in the cognitive ability will show reading deficits only in Language A. Thus, since phonological skills at the phoneme level are important in the reading of English, deficits in those skills might result in developmental dyslexia in English, but not in Japanese. The English-Japanese bilingual case of Wydell and Butterworth (1999), AS, showed poor phonological skills at the phoneme level of English, while his phonological skills at the mora level of Japanese were good. Being parallel with his phonological skills, he showed reading deficits only in English. Similarly, another bilingual boy with developmental dyslexia, CT, also matched the prediction from a cognitive perspective. CT is a Chinese–English biliterate boy. He showed reading deficits in Chinese, but not English. He had impairment in the cognitive abilities required for the development of reading abilities in Chinese, such as phonological skills, naming speed, and orthographic skills in Chinese. In contrast, his phonological skills in English, especially phonemic ones, were good. These cases, AS and CT, suggest that reading impairment in a language emerges when a cognitive ability, which is necessary for reading development in the language, is poor.

### Predictions of literacy profile in a Korean–Japanese bilingual child

Thus far, there has been no report about the dissociation of literacy profiles between Korean and Japanese among bilingual children. However, there are similarities and differences between Korean and Japanese from orthographic and cognitive perspectives. Table 1 summarizes these similarities and differences. Regarding the dissociation of literacy profiles between Korean and Japanese, orthographic and cognitive perspectives hypothesize differently, as follows:

**Table 1:** Summary of the orthographic characteristics and significant the variation of literacy skills among monolingual children.

	Korean (Hangul)	Japanese kana	Japanese kanji
**Orthographic characteristics**			
The number of letters/characters taught in compulsory education	Not many	Not many	Many
Phonological unit corresponding to each letter/character	Phoneme	Mora	Mora ~ Word
Consistency of the conversion from letters/characters to sounds	Consistent	Consistent	Inconsistent
Consistency of the conversion from sounds to letters/characters	Consistent	Consistent	Inconsistent
Visual complexity of each letter/character	Simple	Simple	Complex
**Predictors of reading accuracy of monolinguals**			
Receptive vocabulary	✓	✓	✓
Phonological skills	✓	✓	✓
Naming speed		✓	
Visual skills		✓	✓
**Predictors of spelling/writing accuracy of monolinguals**			
Receptive vocabulary	✓		No data.
Phonological skills	✓	✓	✓
Naming speed		✓	No data.
Visual skills		✓	✓
**Predictors of reading fluency of monolinguals**			
Receptive vocabulary	✓	✓	No data.
Phonological skills	✓	✓	No data.
Naming speed	✓	✓	No data.
Visual skills			No data.

#### Predictions from the orthographic perspective

The Korean writing system is called Hangul, which consists of 14 consonants and 10 vowels. Each letter corresponds to a phoneme. In addition, multiple letters form a block that corresponds to a syllable (e.g., three letters, ㄱ/k/ + ㅗ/o/ + ㅁ/m/ → a syllable, 곰/kom/). The letter-to-sound correspondences are almost consistent. Similarly, the sound-to-letter correspondences are almost consistent as well. Each letter is visually simple.

The Japanese writing system comprises three scripts: hiragana, katakana, and kanji. The former two are called kana. Each kana represents a total of 102 Japanese morae: 46 unvoiced sounds, 18 voiced sounds, 5 p-sounds, and 33 contracted sounds. Characters representing unvoiced sounds are basic. Voiced sounds, p-sounds, and contracted sounds are represented using basic characters. Specifically, the former two are represented by adding a mark to basic characters (e.g., in hiragana, し/shi/ → じ/ji/, は/ha/ → ぱ/pa/; in katakana, シ/shi/ → ジ/ji/, ハ/ha/ → パ/pa/). In contrast, contracted sounds are represented as the combination of multiple characters, whose second-position character is written small (e.g., in hiragana, し/shi/ + よ/yo/ → しょ/sho/; in katakana, シ/shi/ + ヨ/yo/ → ショ/sho/). Like Hangul, the character-to-sound correspondences in kana are almost consistent, irrespective of the direction of conversions between characters and sounds. The phonological unit in Japanese kana is a mora, which is larger than a phoneme. Moreover, each kana character is visually simple because the mean strokes of hiragana and katakana basic characters are 2.3 (SD = 0.9, range: 1–4) and 2.3 (SD = 0.7, range: 1–4), respectively.

Japanese kanji qualitatively differs from Japanese kana. During the 6 years of primary school education, a total of 1,026 kanji characters are learned. Children learn more than 160 kanji characters per year (except in the first year). Each kanji character corresponds to a mora or multiple morae (e.g., the character 医 corresponds to a mora /i/; 学 corresponds to a sound with two morae /gaku/). Several characters can be a word (e.g., the character 犬 is read as /inu/, dog in English). Thus, the phonological unit in Japanese kanji is relatively large. In addition, most kanji characters have multiple pronunciations (e.g., the character 海 has three pronunciations: /umi/ /kai/ and /una/; the character 絵has two pronunciations: /e/ and /kai/). Therefore, the character-to-sound correspondences are inconsistent irrespective of the direction of conversions between characters and sounds. Moreover, kanji is visually more complex than kana because the mean strokes of kanji characters (which are learned by primary school children) are 9.4 (SD = 3.6, range: 1–16).

Although the orthographic characteristics of Japanese kanji differ from those of Korean and Japanese kana, the hypothesis of granularity and transparency (Wydell and Butterworth, 1999) suggests that the writing systems in Korean and Japanese (including kanji) will not cause a high incidence of developmental dyslexia. This is because Hangul and Japanese kana are consistent orthographies (from the transparency dimension). For kanji, this is because its phonological unit is large in Japanese kanji (from the granularity dimension). Thus, the hypothesis predicts that a Korean–Japanese bilingual child tends not to have developmental dyslexia in either language.

#### Predictions from the cognitive perspective

In cohort studies, phonological skills, naming speed, and receptive vocabulary are significant predictors in the development of literacy skills among Korean monolingual children (Cho et al., 2008; Kim, 2008, 2009; Park and Uno, 2012, 2015). According to Park and Uno (2015), phonological skills significantly predict the reading accuracy of first and second graders, while receptive vocabulary predicts the reading accuracy of children in the third grade and above. Moreover, naming speed and phonological skills significantly predict reading fluency among second graders. According to Ju et al.’s (2014) longitudinal study, the reading fluency of children in the second grade was significantly predicted by naming speed, phonological skills, and receptive vocabulary, which were measured in the first grade. Spelling accuracy in Korean monolingual children is significantly predicted by receptive vocabulary (Park and Uno, 2012) and phonological skills (Ju et al., 2016). Importantly, these cohort studies measured children’s visual skills as well. Visual skills are the ability to organize, interpret, and store representations of visual sensory stimuli and their locations. However, visual skills failed to predict the reading and spelling skills of Korean monolingual children (Park and Uno, 2012, 2015; Ju et al., 2016).

In contrast to Korean, visual skills predict reading accuracy in Japanese monolingual children. Regarding Japanese kana, the accuracy of Japanese preschoolers on a hiragana character reading test is significantly predicted by visual skills (Uno et al., 2007) in addition to phonological skills and naming speed (Inomata et al., 2016). Moreover, in addition to receptive vocabulary, phonological and visual skills predict the accuracy of Japanese first graders in hiragana word reading (Hamada and Uno, 2021). As for Japanese kanji, receptive vocabulary and phonological skills are significant predictors of the performance of Japanese primary school children on a kanji word reading test (Uno et al., 2009; Ogino et al., 2017). Additionally, visual skills also predict kanji word reading accuracy among Japanese primary school children (Koyama et al., 2008).

Furthermore, visual skills predict the writing accuracy of Japanese monolingual children. Regarding Japanese kana, in addition to phonological skills and naming speed, visual skills are significant predictors of the accuracy of Japanese preschoolers in hiragana character writing (Inomata et al., 2016). In first graders, phonological and visual skills predict accuracy in hiragana word writing, while naming speed does not (Hamada and Uno, 2021). In subsequent school years, only phonological skills predict accuracy in hiragana word writing (Koyama et al., 2008). Regarding Japanese kanji, phonological, and visual skills predict the accuracy of Japanese primary school children in a kanji word writing test (Koyama et al., 2008). It is unclear whether kanji word writing accuracy can be predicted by naming speed and receptive vocabulary because no studies have investigated this.

In contrast, visual skills do not predict the reading fluency of Japanese monolingual children for any kinds of stimuli (Haruhara et al., 2011). According to Haruhara et al. (2011), phonological skills, naming speed, and receptive vocabulary are significant predictors of children’s speed in reading kana words and text. The former two skills also predict children’s reading speed for kana non-words.

Based on the predictors of monolingual children’s literacy skills in Korean and Japanese, the cognitive perspective can hypothesize the dissociation of literacy skills between the languages in a Korean–Japanese bilingual child, as follows. If a Korean–Japanese bilingual child demonstrates deficits in visual skills apart from deficits in phonological skills and naming speed, the child’s deficits in reading and writing accuracy will be more visible in Japanese. This is because visual skills affect reading and writing accuracy in Japanese but not in Korean. Additionally, given that visual skills are important in learning Japanese kanji relative to Japanese kana (Koyama et al., 2008), the child’s deficits in reading and writing accuracy will be more remarkable in Japanese kanji. However, for reading fluency, the child will display no dissociation between Japanese and Korean because the predictors for reading fluency are common between the languages. To summarize, we hypothesized that a Korean-Japanese bilingual child with low visual skills will show dissociation between the languages in terms of reading and writing accuracy.

### Present study

In the course of our educational consultation activities, we encountered a Korean–Japanese bilingual boy with developmental dyslexia whose literacy profile dissociated between Korean and Japanese in terms of accuracy. In this article, we call him SJ. This article reports on the results of the assessments to measure SJ’s literacy skills, receptive vocabulary, and literacy-related cognitive abilities in Korean and Japanese, using a series of tests to detect developmental dyslexia among monolingual children. The aim of this study was to discuss possible accounts for SJ’s literacy profile, based on the results of the assessments, from orthographic and cognitive perspectives.

Deponio et al. (2000) argued that poor literacy performance can be explained by low socio-economic status or bilingualism, in addition to developmental dyslexia. To rule out bilingualism as a reason for SJ’s low test performances, we compared his test performances to those of Korean–Japanese bilinguals who lived in Japan, in addition to monolinguals. Few case studies have compared test performances of a bilingual child with developmental dyslexia to those of the child’s bilingual peers.

## Materials and methods

There are no tests for examining developmental dyslexia in Korean and Japanese among Korean–Japanese bilingual children. For Korean, we used the tests that were used by the second author and her colleagues (e.g., Park and Uno, 2010, 2012, 2015; Ju et al., 2014, 2016). For Japanese, we used a series of tests that were used for the diagnosis of Japanese developmental dyslexia (e.g., Sambai et al., 2016; Uno et al., 2018). These tests measured SJ’s reading and writing abilities, receptive vocabulary, and cognitive abilities (i.e., phonological skills, naming speed, and visual skills).

### Participants

#### Case history of SJ

At the time of testing, a Korean–Japanese bilingual boy, SJ, was in the sixth grade of a primary school (at the age of 11). He was born to Korean parents in Japan. He lived in Korea from ages two to four and has lived in Japan thereafter. Since his birth, he and his family have been using Korean to communicate with each other.

SJ enrolled in a private kindergarten in Japan at the age of four. In kindergarten, Japanese was used for all activities. However, he could not speak and aurally understand Japanese well because he used Korean at home. He started to learn Japanese through activities with his Japanese kindergarten classmates.

Later, SJ entered a private primary school in Japan at the age of six. SJ and his classmates received bilingual education in Japanese and Korean. The primary school students learned all subjects, except for Korean language classes, in Japanese, following the national curriculum formulated by the Ministry of Education, Culture, Sports, Science, and Technology in Japan. Most students and teachers had roots in Korea, irrespective of their nationality and the predominant languages used at home. Korean language classes were organized according to the students’ Korean language skills. SJ studied Korean in a superior Korean language class, alongside students whose first language (L1) was Korean.

At 11, in the sixth grade, SJ’s parents and teacher contacted us in 20XX as they were concerned about his literacy skills. They informed us of his difficulty in learning to read and write in Japanese, especially Japanese kanji, despite no issues with his oral communication in Japanese. The teacher was also concerned about his reading speed in both Japanese and Korean. Therefore, we conducted assessments in Korean and Japanese to examine whether his reading and writing difficulties resulted from bilingualism or developmental dyslexia. Thereafter, he was diagnosed with a specific learning disorder at the age of 13 when he was in the second grade of junior high school.

We measured SJ’s general intelligence with the Japanese version of the Wechsler Intelligence Scale for Children Fourth Edition (WISC-IV; Ueno et al., 2010) and Raven’s Colored Progressive Matrices (RCPM; Raven, 1976) when he was in the sixth grade of primary school (at the age of 11). Table 2 shows SJ’s general intelligence test performances. His Full Scale IQ (FSIQ) of WISC-IV was low average (his FSIQ = 81). However, his Perceptual Reasoning Index (PRI) of WISC-IV and his score on the RCPM (as a non-verbal intelligence test) were within the average range of Japanese monolingual children (Uno et al., 2017). Thus, we considered that at least his nonverbal intelligence was within the average level.

**Table 2:** SJ’s general intelligence.

	SJ’s performance (age 11)			Japanese monolingual children	
		Comparison with the norm of Japanese monolinguals		Mean	SD
**WISC-IV**					
FSIQ	81	Average	< −1.0 SD	100	15
VCI	80	Average	< −1.0 SD	100	15
PRI	93	Average	Within ±1.0 SD	100	15
WMI	73	Low	< −1.5 SD	100	15
PSI	91	Average	Within ±1.0 SD	100	15
**RCPM**					
Score (max = 36)	31	Average	Within ±1.0 SD	33.0	3.8

For the WISC-IV, each SJ’s score was a standard score; the norm of Japanese monolinguals was obtained from Ueno et al. (2010). For RCPM, SJ’s score was the number of correct responses; the norm of Japanese monolinguals was obtained from Uno et al. (2017).

The Japanese version of the attention deficit hyperactivity disorder (ADHD) Rating Scale-IV (Ichikawa and Tanaka, 2008) was conducted for screening for ADHD. For this questionnaire, SJ’s mother was the informant. SJ’s scores met the criteria to rule out the possibility of ADHD (his ADHD-RS total score = 13, his inattention subscale score = 9, and his hyperactivity-impulsivity subscale score was 4). Moreover, the imitation of hand and finger postures in the Standard Performance Test of Apraxia (Japan Society for Higher Brain Dysfunction, 1985) was used to assess SJ’s fine motor movement. Specifically, SJ was asked to see a tester’s fingers and make the same shapes (e.g., fox and pigeon). In addition, he was asked to quickly open and close the left and right hands alternately five times. He showed no clumsiness on these tests. Thus, SJ likely had neither ADHD nor clumsiness.

#### Bilingual participants for the assessment in Korean

For SJ, Korean is the first language (L1), but it is a heritage language in Japan. Therefore, he might not receive a sufficient amount of input in Korean. Consequently, he might not develop his receptive vocabulary and literacy skills in Korean as sufficiently as Korean monolingual children of the same age. SJ had learned to read and write in Hangul with less than 10 Korean–Japanese bilingual classmates whose L1 was Korean. Educational circumstances in learning Korean were similar between SJ and his bilingual classmates since they learned it from the same teachers using the same textbooks. Therefore, SJ’s test performances in Korean were compared to those of his bilingual classmates.

Eight of SJ’s classmates (three boys and five girls) participated in this study in 20XX. They were all Korean–Japanese bilingual children in the sixth grade of primary school. Their ages were between 11 and 12 years old. The test score of each participant on the RCPM (a non-verbal intelligence test) was higher than −1.5 SD from the mean of Japanese monolingual children (Uno et al., 2017). This means that their scores were within the average range, like that of SJ. Their teachers confirmed that none had difficulties in reading and writing in both Korean and Japanese, based on their observations of school learning activities.

#### Bilingual participants for the assessment in Japanese

We could not carry out the data collection of Korean–Japanese bilingual controls in the assessment in Japanese in 20XX (i.e., the year when SJ took tests) due to time constraints in the school schedule. Therefore, we recruited Korean–Japanese bilingual children in the sixth grade (11 and 12 years old) from the same school as SJ and administered tests in 20XX + 2 and 20XX + 3 (after SJ graduated from primary school). Ten bilingual children participated in this study in 20XX + 2, and eight bilingual children participated in 20XX + 3. Thus, a total of 18 bilingual children (10 boys and 8 girls) participated.

Since SJ’s non-verbal intelligence was within the average level, we excluded from the analyses, three participants whose test scores on the RCPM (a non-verbal intelligence test) were below 1.5 SD from the mean of Japanese monolingual children (Uno et al., 2017). Consequently, data on 15 bilingual children (7 boys and 8 girls) were used to compare with SJ’s test performance. Their teachers confirmed that none had difficulties in reading and writing in Japanese, based on their observations of school learning activities.

### Tests for the assessment in Korean

The following tests were the same as Park and Uno’s (2015) study. Each receptive vocabulary and cognitive test had a few practice trials. Since SJ and bilingual participants responded to the practice trials correctly on each test, we confirmed that they understood the instructions of each test.

SJ and the bilingual participants completed all the tests at their school on the same day, using the same procedure. Children took the tests in both group and individual test sessions. The group test session, which lasted about 30 min, assessed participants’ receptive vocabulary and spelling skills. The individual test session, which lasted about 20 min in a quiet classroom, assessed participants’ reading skills, phonological skills, and naming speed.

#### Reading and spelling skills in Korean

Word and non-word reading tests were used to assess reading accuracy in Korean. The tests comprised 23 words and 5 non-words. The number of correct responses was recorded per test.

Reading fluency in Korean was measured by rapid reading-aloud tests. The tests had three sets of stimuli: words, non-words, and a paragraph. In each set, children were asked to read aloud as quickly and accurately as possible. The time required to finish reading (RTs) was measured by a stopwatch per set.

A spelling test was administered to assess spelling accuracy in Korean. The test comprised seven words and three non-words. Children were asked to write each orally presented stimulus in Hangul, and the number of correct responses was recorded.

#### Receptive vocabulary in Korean

We assessed receptive vocabulary in Korean, using the receptive vocabulary subtest of the Receptive and Expressive Vocabulary Test (REVT; Kim et al., 2009), which was shortened by Park and Uno (2015). The shortened version consists of 23 trials. Multiple pictures were provided, and each target word was presented orally. Children were asked to select one corresponding picture per presented word. The number of correct responses was recorded.

#### Phonological skills in Korean

To assess phonological skills in Korean, we used a series of phonological tests. The tests consisted of non-word repetition, syllable deletion, phoneme onset deletion, phoneme coda deletion, and phoneme onset oddity tests. The number of correct responses was recorded per test.

The non-word repetition test included seven stimuli with 4 to 10 syllables. Children were asked to listen to each non-word carefully and then repeat it. Seven trials were conducted after one practice trial.

In the syllable deletion test, children were asked to delete the syllable of an orally presented word in the indicated position (i.e., the beginning-syllable position, the middle-syllable position, or, the final-syllable position). Then, they were required to answer the remaining sound. Five trials were conducted after two practice trials. Three trials were classified into the middle-syllable position condition. The remaining trials (*n =* 2) were classified into the first-syllable or the final-syllable position conditions.

In the phoneme onset and coda deletion tests, children were asked to delete the onset or coda of an orally presented monosyllabic or two-syllable word and then answer the remaining sound. Each test consisted of two practice trials and five trials.

In the phoneme onset oddity test, three monosyllabic words were presented orally per trial. Children were asked to say the word whose initial phoneme (i.e., onset) differed from that of the others. Five trials were conducted after two practice trials.

#### Naming speed in Korean

We measured naming speed in Korean with the rapid automatized naming test (RAN) which consisted of one practice trial and three trials. For each trial, drawings and digits were alternately arranged in four rows of five each on an A4 paper. Children were asked to say the names of stimuli in each row as quickly as possible in Korean. For every trial, the time required to finish a response (RTs) was measured using a stopwatch and the mean RTs of three trials were calculated.

### Tests for the assessment in Japanese

All tests were administered to SJ face-to-face across four sessions over 3 weeks. Each session lasted between one and 2 h. Unfortunately, we could not conduct the data collection of bilingual participants using the same tests as for SJ due to time constraints in the school schedule. The bilingual participants took only a part of the tests that were administered to SJ (some of which were shortened). The testing was conducted in both group and individual test sessions. The group test session lasted 40 min and assessed each participant’s receptive vocabulary and writing skills, as well as visual skills. The individual test session lasted between 15 and 20 min in a quiet classroom and assessed each participant’s reading skills, phonological skills, and naming speed.

Each receptive vocabulary and cognitive test had a few practice trials. Since SJ and the bilingual participants responded correctly to the practice trials for each test, we confirmed that they understood the instructions of each test.

#### Reading skills in Japanese

We assessed SJ’s reading accuracy in Japanese kana, using four subtests of the Standardized Test for Assessing the Reading and Writing (Spelling) Attainment of Japanese Children and Adolescents: Accuracy and Fluency (STRAW-R; Uno et al., 2017): (i) the hiragana character reading test, (ii) the katakana character reading test, (iii) the hiragana word reading test for sixth graders, and (iv) the katakana word reading test for sixth graders. All subtests were standardized among Japanese monolingual children. Each test consisted of 20 stimuli. The number of correct responses was recorded per test.

Regarding reading accuracy in Japanese kanji, three tests were administered to SJ: (i) the kanji word-reading test for sixth graders in STRAW-R (Uno et al., 2017), (ii) the 126 kanji word reading test in STRAW-R (Uno et al., 2017), and (iii) the word reading test in the Kaufman Assessment Battery for Children Second Edition (KABC-II; Fujita et al., 2013). All tests were standardized among Japanese monolingual children. The kanji word reading test for sixth graders in STRAW-R consisted of 20 kanji words. In the 126 kanji word reading test of STRAW-R and the word reading test of KABC-II, the difficulty level of stimuli progressively increased. Both tests can estimate the child’s reading age based on the number of correct responses. The number of correct responses was recorded per test.

In addition, SJ’s reading fluency in Japanese was measured by the rapid reading-aloud tests of STRAW-R (Uno et al., 2017). The tests were standardized among Japanese monolingual children. They consist of five sets of stimuli: hiragana words, katakana words, hiragana non-words, katakana non-words, and text. In each set, SJ was asked to read aloud as quickly and accurately as possible. The time required to finish reading (RTs) was measured by a stopwatch per set.

No tests to assess reading accuracy in Japanese kana were administered to the bilingual participants since they were expected to show the ceiling effect in each test and owing to time constraints in the school schedule. We measured their reading accuracy in Japanese kanji and reading fluency using the 126 kanji word reading test and the rapid reading-aloud tests of STRAW-R (Uno et al., 2017), respectively.

#### Writing skills in Japanese

We measured SJ’s writing accuracy in Japanese kana with four subtests of STRAW-R (Uno et al., 2017): (i) the hiragana character writing test, (ii) the katakana character writing test, (iii) the hiragana word writing test for sixth graders, and (iv) the katakana word writing test for sixth graders. All tests were standardized among Japanese monolingual children. Each subtest had 20 stimuli. SJ was required to write an orally presented sound in both hiragana and katakana. The number of correct responses was recorded per test.

We measured SJ’s writing accuracy in Japanese kanji with two tests: (i) the kanji word writing test for sixth graders in STRAW-R (Uno et al., 2017) and (ii) the word writing test in KABC-II (Fujita et al., 2013). Both tests were standardized among Japanese monolingual children. The kanji word writing test of STRAW-R consisted of 20 stimuli. In the word writing test of KABC-II, the difficulty level of stimuli progressively increased and SJ’s writing age was estimated based on the number of correct responses. In both tests, SJ was asked to write an orally presented word in kanji. The number of correct responses was recorded per test.

The bilingual participants took only three tests, the hiragana, katakana, and kanji word writing tests for sixth graders in STRAW-R.

#### Receptive vocabulary in Japanese

We measured SJ’s receptive vocabulary in Japanese with three standardized tests: (i) the Picture Vocabulary Test-Revised (PVT-R; Ueno et al., 2008), (ii) the Standardized Comprehension Test of Abstract Words (SCTAW; Uno et al., 2002), and (iii) verbal knowledge in KABC-II (Fujita et al., 2013). Only SCTAW, shortened by Sano et al. (2017), was administered to the bilingual participants.

In the PVT-R and the verbal knowledge test of KABC-II, the difficulty level of stimuli in each test progressively increased and SJ’s vocabulary age was estimated based on the number of correct responses. The full version of SCTAW consisted of 45 trials, while its shortened version had 16 trials. In all the tests, multiple pictures were provided, and each target word was presented orally. Children were asked to select one corresponding picture per presented word. The number of correct responses was recorded per test.

#### Phonological skills in Japanese

The non-word repetition test used by Uno et al. (2018), was administered to SJ and the bilingual participants. The test consisted of 10 stimuli with four to nine morae. One practice trial and 10 trials were conducted. Children were asked to listen to each non-word carefully and then repeat it. The number of correct responses was recorded.

In addition, we used the word backward span test (Uno et al., 2018) to measure SJ’s phonological skills at the mora level. The stimuli consisted of 20 words with three or four morae (for each, 10). Due to time constraints, a shortened version of the word backward span test was administered to the bilingual participants. Out of the stimuli with four morae, five were used in the shortened version. For each trial, children were asked to listen to a word carefully and repeat it. After accurate repetition, they were asked to repeat the word in reverse (e.g., “ka-mi-na-ri” → “ri-na-mi-ka”). The number of correct responses was recorded. Moreover, the time required to finish a response (RTs) was measured with a stopwatch. The mean RTs for the correct responses were calculated.

#### Naming speed in Japanese

The rapid automatized naming (RAN) test in the STRAW-R (Uno et al., 2017) was administered to SJ and bilingual participants to assess naming speed in Japanese. One practice trial and three trials were conducted. For each trial, drawings, and digits were alternately arranged in four rows of five each on an A4 paper. Children were asked to say the names of stimuli in each row as quickly as possible in Japanese. For every trial, the time required to finish a response (RTs) was measured using a stopwatch. The mean RTs of the three trials were calculated.

#### Visual skills

We measured SJ’s visual skills with the Matching Familiar Figure Test (MFFT; Kaneko et al., 2020) and the Rey-Osterrieth Complex Figure Test (ROCFT; Rey, 1941). Only ROCFT was administered to the bilingual participants.

MFFT had 12 trials. In each trial, SJ was asked to examine six line-drawings carefully and point to the same line-drawing as the target. The distractors resembled the target visually. Trials lasted until he found the correct line-drawing. The number of correct responses and pointing to the distractors was recorded. The response latency (RTs) was measured by a stopwatch per trial.

The ROCFT comprised three tests: copy drawing, immediate recall, and delayed recall tests. Children were asked to copy a target figure (copy drawing) and reproduce the figure immediately (immediate recall) and after 30 min (delayed recall). Each test score was calculated.

### Definition of low performance on each test

SJ’s test performances were compared to the previously published norm of monolingual children and the performances of bilingual participants. The norm of Korean monolingual children was obtained from Park and Uno’s (2015) study, since this study used their tests for the assessment in Korean. Unfortunately, Park and Uno (2015) did not collect data on Korean monolingual children in the sixth grade. Therefore, SJ’s test performance on each test was compared to the norm of their oldest participants, namely, fourth graders (aged nine or 10 years). Regarding the assessment in Japanese, the previously published norm of Japanese monolingual children in the sixth grade was used. If SJ’s performance was below 1.5 SD from the mean of monolinguals, we defined his performance as ‘low.’ Otherwise, we referred to his performance as ‘within the average range.’

Crawford and Howell (1998) argued that, in a case study, the *t*-test should be used when the number of the normative sample is less than 50. The number of our bilingual participants was small. Therefore, we performed the modified *t*-test (Crawford and Howell, 1998) when we compared SJ’s test performances to those of the bilinguals. If his performance was significantly lower than that of bilingual participants, we defined his performance as ‘low.’

## Results

### Assessment in Korean

Table 3 shows SJ’s performances on the reading and spelling tests in Korean, and Table 4 shows his performances on the tests that assessed receptive vocabulary and cognitive abilities in Korean. Table 5 shows the individual performances of bilingual participants on those tests.

**Table 3:** SJ’s reading and spelling skills in Korean.

	SJ’s performance (Age 11)				Korean monolingual children		Bilingual participants		
		Comparison							
		SJ vs. monolinguals		SJ vs. bilinguals	Mean	SD	Mean	SD	Range
**Reading accuracy in Korean**									
Word and non-word reading tests									
Word scorem (max = 23)	9	Low	< −1.5 SD	*t*(7) = −0.819, *p* = 0.440	13.9	2.5	12.38	4.09	5–18
Non-word score (max = 5)	3	Low	< −3.5 SD	*t*(7) = −1.875, *p* = 0.103	4.8	0.5	4.25	0.66	3–5
**Reading fluency in Korean**									
Rapid reading aloud tests									
Words RTs (in second)	51.91	Low	< −16.0 SD	*t*(7) = 19.303, *p* < 0.001	11.0	2.5	10.82	2.11	8.64–15.76
Non-words RTs (in seconds)	61.96	Low	< −8.0 SD	*t*(7) = 12.766, *p* < 0.001	20.4	5.0	17.46	3.46	11.96–21.43
Paragraph RTs (in seconds)	242.7	Low	< −14.0 SD	*t*(7) = 22.373, *p* < 0.001	60.9	12.7	53.08	8.41	44.39–69.68
**Spelling accuracy in Korean**									
Spelling test									
Score (max = 10)	4	Average	within ±1.0SD	*t*(7) = −0.285, *p* = 0.784	5.5	1.7	4.63	2.18	1–9

For each test, the norm of Korean monolinguals was obtained from Park and Uno (2015).

**Table 4:** SJ’s receptive vocabulary and cognitive abilities in Korean.

	SJ’s performance (Age 11)				Korean monolingual children		Bilingual participants		
		Comparisons							
		SJ vs. monolinguals		SJ vs. bilinguals	Mean	SD	Mean	SD	Range
**Receptive vocabulary in Korean**									
Receptive vocabulary test									
Score (max = 23)	13	Average	< −1.0 SD	*t*(7) = −0.261, *p* = 0.802	16.2	2.9	14.00	3.81	6–18
**Phonological skills in Korean**									
Non-word repetition test									
Score (max = 7)	3	Average	Within ±1.0SD	*t*(7) = −0.143, *p* = 0.196	3.5	1.3	4.13	0.78	3–5
Syllable deletion test									
Score (max = 5)	5	Average	Within ±1.0SD		4.9	0.3	5.00	0.00	5–5
Phoneme onset deletion test									
Score (max = 5)	5	Average	Within ±1.0SD	*t*(7) = 0.375, *p* = 0.719	4.4	1.0	4.88	0.33	4–5
Phoneme coda deletion test									
Score (max = 5)	4	Average	Within ±1.0SD	*t*(7) = −2.625, *p* = 0.034	4.7	0.8	4.88	0.33	4–5
Phoneme onset oddity test									
Score (max = 5)	2	Low	< −2.0 SD	*t*(7) = −3.742, *p* = 0.007	4.3	1.0	4.63	0.70	3–5
Combined phoneme manipulation tests									
Score (max = 15)	11			*t*(7) = −4.811, *p* = 0.002			14.38	0.70	13–15
**Naming speed in Korean**									
Rapid automatized naming test (RAN)									
RTs (in seconds)	18.27	Low	< −2.5 SD	*t*(7) = 8.894, *p* < 0.001	12.1	2.3	9.82	0.94	7.83–10.84

For each test, the norm of Korean monolinguals was obtained from Park and Uno (2015).

**Table 5:** Test performances of each bilingual participant in the assessment in Korean.

	1	2	3	4	5	6	7	8
	Boy	Boy	Boy	Girl	Girl	Girl	Girl	Girl
**Reading accuracy in Korean**								
Word and non-word reading tests								
Word score (max = 23)	11	12	5	18	14	9	12	18
Non-word score (max = 5)	4	5	4	5	4	3	4	5
**Reading fluency in Korean**								
Rapid reading aloud tests								
Words RTs (in second)	11.2	8.64	11.43	10.55	10.72	15.76	9.54	8.75
Non-words RTs (in second)	20.61	16.02	20.44	11.96	20.36	21.43	15.38	13.45
Paragraph RTs (in second)	48.33	47.56	69.68	44.39	57.17	62.17	46.30	49.05
**Spelling accuracy in Korean**								
Spelling test								
Score (max = 10)	6	4	1	9	4	3	5	5
**Receptive vocabulary in Korean**								
Receptive vocabulary test								
Score (max = 23)	18	17	10	15	15	14	6	17
**Phonological skills in Korean**								
Non-word repetition test								
Score (max = 7)	4	3	4	5	3	4	5	5
Syllable deletion test								
Score (max = 5)	5	5	5	5	5	5	5	5
Phoneme onset deletion test								
Score (max = 5)	5	5	5	5	5	4	5	5
Phoneme coda deletion test								
Score (max = 5)	4	5	5	5	5	5	5	5
Phoneme onset oddity test								
Score (max = 5)	5	4	3	5	5	5	5	5
Combined Scores of the three phoneme manipulation tests								
Score (max = 15)	14	14	13	15	15	14	15	15
**Naming speed in Korean**								
Rapid automatized naming test (RAN)								
RTs (in seconds)	10.08	9.69	10.75	9.38	10.67	10.84	9.34	7.83

Regarding reading and spelling skills in Korean, SJ showed low performances on the word and non-word reading tests and on each set of the rapid reading-aloud tests, compared to Korean monolinguals. In contrast, his performance on the spelling test was within the average range of Korean monolinguals. In the modified *t*-test, SJ’s performances on only the rapid reading-aloud tests were significantly lower than those of bilingual participants.

Out of receptive vocabulary and cognitive tests, SJ showed low performances on the phoneme onset oddity test and the RAN test, compared to Korean monolinguals. In the comparison with bilingual participants, SJ’s performances were significantly lower on the phoneme coda deletion test, phoneme oddity test, and the RAN test. The former two measured SJ’s phonological skills at the phoneme level. Therefore, SJ’s phonological skills, especially at the phoneme level, appeared to be poor. However, each phonological test consisted of only five trials. To confirm this, we performed the modified t-test on the combined score of three phonological tests at the phoneme level (i.e., the phoneme onset deletion test, the phoneme coda deletion test, and the phoneme oddity test). As a result, his combined score was significantly lower than that of bilinguals.

### Assessment in Japanese

Table 6 shows SJ’s performances on the reading and writing tests in Japanese. Table 7 shows his performances on the tests to assess receptive vocabulary and cognitive abilities in Japanese. Table 8 shows the individual performances of bilingual participants on those tests.

**Table 6:** SJ’s reading and writing skills in Japanese.

	SJ’s performance (age 11)				Japanese monolingual children		Bilingual participants		
		Comparisons							
		SJ vs. monolinguals		SJ vs. bilinguals	Mean	SD	Mean	SD	Range
**Reading accuracy in Japanese**									
Hiragana and Katakana character reading tests of STRAW-R									
Hiragana score (max = 20)	20	Average	Within ±1.0 SD		19.9	0.2			
Katakana score (max = 20)	19	Low	−3.0 SD		19.9	0.3			
Word reading tests for sixth graders in STRAW-R									
Hiragana score (max = 20)	18	Low	< −4.5 SD		19.9	0.4			
Katakana score (max = 20)	19	Low	< −1.5 SD		19.9	0.5			
Kanji Score (max = 20)	3	Low	−6.0 SD		19.2	2.7			
126 kanji word reading test of STRAW-R									
Score (max = 126)	31	Low	< −6.0 SD	*t*(14) = −5.37, *p* < 0.001	113.4	12.7	108.26	14.36	71–123
Age equivalent (years)	7:09								
Word reading test of KABC-II									
Scaled score	3	Low	< −2.0 SD		10	3			
Age equivalent (years)	7:09								
**Reading fluency in Japanese**									
Rapid reading-aloud tests of STRAW-R									
Hiragana words RTs (in seconds)	104	Low	< −24.0 SD	*t*(14) = 10.94, *p* < 0.001	16.59	3.63	19.44	7.74	10.14–39.42
Katakana words RTs (in seconds)	133	Low	< −32.5 SD	*t*(14) = 17.52, *p* < 0.001	15.70	3.59	18.57	6.52	10.10–34.91
Hiragana non-words RTs (in seconds)	100	Low	< −14.5 SD	*t*(14) = 12.35, *p* < 0.001	21.78	5.33	20.63	6.41	8.59–34.70
Katakana non-words RTs (in seconds)	100	Low	< −13.0 SD	*t*(14) = 14.37, *p* < 0.001	21.43	5.89	20.31	5.53	9.26–30.11
Text RTs (in seconds)	295	Low	< −21.0 SD	*t*(14) = 10.73, *p* < 0.001	51.38	11.35	57.83	22.06	32.22–119.38
**Writing accuracy in Japanese**									
Hiragana and Katakana character writing tests of STRAW-R									
Hiragana score (max = 20)	19	Average	< −1.0 SD		19.8	0.6			
Katakana score (max = 20)	17	Average	Within ±1.0 SD		19.2	2.7			
Word writing tests for sixth graders in STRAW-R									
Hiragana score (max = 20)	16	Low	< −1.5 SD	*t*(14) = −7.00, *p* < 0.001	19.7	2.0	19.8	0.54	18–20
Katakana score (max = 20)	7	Low	< −5.0 SD	*t*(14) = −2.41,*p* = 0.030	19.2	2.4	18.2	4.64	3–20
Kanji Score (max = 20)	1	Low	< −2.5 SD	*t*(14) = −5.80, *p* < 0.001	15.0	5.4	15.93	2.57	11–20
Word writing test of KABC-II									
Scaled score	3	Low	< −2.0 SD		10	3			
Age equivalent (years)	7:06								

For each subtest of STRAW-R, the norm of Japanese monolinguals was obtained from Uno et al. (2017). For the subtest of KABC-II, the norm of Japanese monolinguals was obtained from Fujita et al. (2013). Score of each test means the number of correct responses.

**Table 7:** SJ’s receptive vocabulary and cognitive abilities in Japanese.

	SJ’s performance (Age 11)				Japanese monolingual children		Bilingual participants		
		Comparisons							
		SJ vs. monolinguals		SJ vs. bilinguals	Mean	SD	Mean	SD	Range
**Receptive vocabulary in Japanese**									
PVT-R									
Scaled score	9	Average	Within ±1.0 SD		10	3			
Vocabulary age (years)	10:04								
SCTAW									
Full version score (max = 45)	28	Average	< −1.0 SD		33.40	4.50			
Shortened version score (max = 16)	8	Low	< −1.5 SD	*t*(14) = −2.50, *p* = 0.025	12.57	2.44	11.93	1.57	9–15
Verbal knowledge test of KABC-II									
Scaled Score	9	Average	Within ±1.0 SD		10	3			
Age equivalent (years)	10:00								
**Phonological skills in Japanese**									
Non-word repetition test									
Score (max = 10)	6	Low	< −1.5 SD	*t*(14) = −1.67, *p* = 0.118	7.96	1.27	8.07	1.24	6–10
Word backward span test [the full version]									
Correct rates (%)	55	Average	< −1.0 SD		76.92	16.92			
RTs of three-mora words (in seconds)	7.55	Low	< −2.5 SD		2.9	1.7			
RTs of four-mora words (in seconds)	22.75	Low	< −3.5 SD		6.6	4.3			
Word backward span test [the shortened version]									
Score (max = 5)	3			*t*(14) = −1.90, *p* = 0.078			4.33	0.70	3–5
RTs (in seconds)	24.96			*t*(14) = 7.41, *p* < 0.001			4.07	2.81	1.41–10.97
**Naming speed in Japanese**									
RAN test									
RTs (in seconds)	24.54	Low	< −7.0 SD	*t*(14) = 8.12, *p* < 0.001	10.3	2.0	10.12	1.77	6.47–13.19
**Visual skills**									
MFFT									
Score (max = 12)	5	Average	Within ±1.0SD		7.2	2.5			
Number of pointing the distractors	11	Average	Within ±1.0SD		6.8	4.6			
RTs (in seconds)	24.7	Average	Within ±1.0SD		19.4	10.4			
ROCFT									
Copy drawing score (max = 36)	22	Low	< −2.5 SD	*t*(14) = −3.91, *p* = 0.002	32.7	3.7	32.50	2.68	27–35
Immediate recall score (max = 36)	17.5	Average	Within ±1.0SD	*t*(14) = −0.951, *p* = 0.358	22.8	5.9	24.57	7.41	8–35
Delayed recall score (max = 36)	17.5	Average	−1.0 SD	*t*(14) = −1.39, *p* = 0.187	23.3	5.8	26.07	6.16	15–35

For the PVT-R, the norm of Japanese monolinguals was obtained from Ueno et al. (2008). For SCTAW, the norm of Japanese monolinguals in the full version was obtained from Uno et al. (2002), while the norm of Japanese monolinguals in the shortened version was obtained from Sano et al. (2017). For the subtest of KABC-II, the norm of Japanese monolinguals was obtained from Fujita et al. (2013). For tests related to phonological and visual skills, the norm of Japanese monolinguals was obtained from Uno et al. (2018). For the RAN test, the norm of Japanese monolinguals was obtained from Uno et al. (2017).

**Table 8:** Test performances of each bilingual participant in the assessment in Japanese.

	1	2	3	4	5	6	7	8	9	10	11	12	13	14	15
	Boy	Boy	Boy	Boy	Boy	Boy	Boy	Girl	Girl	Girl	Girl	Girl	Girl	Girl	Girl
**Reading and writing accuracy**															
126 kanji word reading test in STRAW-R															
Score (max = 126)	90	101	71	115	123	120	94	123	98	120	116	106	112	116	119
Word writing tests for sixth graders in STRAW-R															
Hiragana score (max = 20)	20	20	18	20	20	20	20	20	20	20	20	20	19	20	20
Katakana score (max = 20)	11	20	3	20	20	20	20	20	20	20	20	19	20	20	20
Kanji score (max = 20)	11	18	12	16	20	20	13	16	17	16	15	15	19	15	16
**Reading fluency in Japanese**															
Rapid reading-aloud tests in STRAW-R															
Hiragana words RTs (in seconds)	39.42	20.08	35.68	18.06	13.361	14.26	18.93	10.14	19.48	18.22	15.94	21.86	14.94	12.96	18.33
Katakana words RTs (in seconds)	34.91	19.71	31.29	19.62	13.02	16.52	16.96	10.1	19.37	16.76	14.38	22.44	15.21	11.98	16.24
Hiragana non-words RTs (in seconds)	34.7	25.86	29.1	18.92	12.44	22.45	21.67	8.59	24.81	14.23	18.24	20.95	15.77	18.39	23.37
Katakana non-words RTs (in seconds)	30.11	26.6	25.59	20.66	11.76	19.81	21.08	9.26	24.41	17.61	18.06	26.57	16.51	16.25	20.42
Text RTs (in seconds)	94.73	60.41	119.38	53.54	38.58	45.69	69.36	32.22	58.34	45.45	52.48	62.95	48.07	36.66	49.64
**Receptive vocabulary in Japanese**															
SCTAW (the shortened version)															
Score (max = 16)	12	12	14	11	9	12	12	14	12	15	12	11	13	10	10
**Phonological skills in Japanese**															
Non-word repetition test															
Score (max = 10)	8	8	9	7	10	9	7	9	10	9	8	6	6	8	7
Word backward span test (the shortened version)															
Score (max = 5)	3	5	4	5	4	5	5	5	4	5	3	4	5	4	4
RTs (in seconds)	7.81	2.07	7.17	4.11	1.41	6.75	1.94	1.47	5.59	2.30	1.75	10.97	2.04	2.91	2.83
**Naming speed**															
RAN test															
RTs (in seconds)	13.187	10.09	12.197	10.3	9.0433	9.7067	9.0933	6.47	10.647	11.923	11.887	9.1	7.76	8.69	11.64
**Visual skills**															
ROCFT															
Copy drawing score (max = 36)	33	29	27	35	35	32.5	35	35	33	32	27	32	33	35	34
Immediate recall score (max = 36)	27	22	25	31	29	35	30	22	25	8	13.5	22	15	32	32
Delayed recall score (max = 36)	28	28	28	30	33	35	29	24	21	15	16	22	18	32	32

Uno et al. (2009) reported significant gender differences in the prevalence of reading and writing difficulties in Japanese. Therefore, SJ’s performances were compared to bilingual boys and girls separately, in addition to the comparison with the mixture of bilingual boys and girls. Table 9 shows the comparisons of SJ’s test performances with those of bilingual participants per gender.

**Table 9:** Comparisons of SJ’s performances with those of bilingual boys and girls in the assessment in Japanese.

	Bilingual Boys (n = 7)			Bilingual Girls (n = 8)		
	Mean	SD	SJ vs. Bilingual boys	Mean	SD	SJ vs. Bilingual girls
**Reading and writing accuracy**						
126 kanji word reading test in STRAW-R						
Score (max = 126)	102.00	17.34	*t*(6) = −4.05, *p* = 0.007	113.75	7.69	*t*(7) = −10.67, *p* < 0.001
Word writing tests for sixth graders in STRAW-R						
Hiragana score (max = 20)	19.71	0.70	*t*(6) = −5.25, *p* = 0.002	19.88	0.33	*t*(7) = −11.63, *p* < 0.001
Katakana score (max = 20)	16.29	6.25	*t*(6) = −1.47, *p* = 0.192	19.88	0.33	*t*(7) = −38.63, *p* < 0.001
Kanji score (max = 20)	15.71	3.49	*t*(6) = −4.17, *p* = 0.006	16.13	1.27	*t*(7) = −11.83, *p* < 0.001
**Reading fluency**						
Rapid reading-aloud tests in STRAW-R						
Hiragana words RTs (in seconds)	22.83	9.63	*t*(6) = 8.34, *p* < 0.001	16.48	3.53	*t*(7) = 24.85, *p* < 0.001
Katakana words RTs (in seconds)	21.72	7.55	*t*(6) = 10.78, *p* < 0.001	15.81	3.66	*t*(7) = 23.88, *p* < 0.001
Hiragana non-words RTs (in seconds)	23.59	6.66	*t*(6) = 11.36, *p* < 0.001	18.04	4.90	*t*(7) = 16.60, *p* < 0.001
Katakana non-words RTs (in seconds)	22.23	5.50	*t*(6) = 14.00, *p* < 0.001	18.64	4.99	*t*(7) = 16.19, *p* < 0.001
Text RTs (in seconds)	68.81	26.68	*t*(6) = 8.39, *p* < 0.001	48.23	9.60	*t*(7) = 25.51, *p* < 0.001
**Receptive vocabulary**						
SCTAW (the shortened version)						
Score (max = 16)	11.71	1.39	*t*(6) = −2.65, *p* = 0.038	12.13	1.69	*t*(7) = −2.43, *p* = 0.046
**Phonological skills**						
Non-word repetition test						
Score (max = 10)	8.29	1.03	*t*(6) = −2.20, *p* = 0.070	7.88	1.36	*t*(7) = −1.36, *p* = 0.215
Word backward span test (the shortened version)						
Score (max = 5)	4.43	0.73	*t*(6) = −1.94, *p* = 0.100	4.25	0.66	*t*(7) = −1.88, *p* = 0.103
RTs (in seconds)	4.47	2.54	*t*(6) = 7.97, *p* < 0.001	3.73	2.98	*t*(7) = 7.06, *p* < 0.001
**Naming speed**						
RAN test						
RTs (in seconds)	10.52	1.47	*t*(6) = 9.47, *p* < 0.001	9.76	1.93	*t*(7) = 7.58, *p* < 0.001
**Visual skills**						
ROCFT						
Copy drawing score (max = 36)	32.36	2.96	*t*(6) = −3.46, *p* = 0.013	32.63	2.39	*t*(7) = −4.40, *p* = 0.003
Immediate recall score (max = 36)	28.43	3.92	*t*(6) = −2.76, *p* = 0.033	21.19	8.07	*t*(7) = −0.45, *p* = 0.664
Delayed recall score (max = 36)	30.14	2.59	*t*(6) = −4.84, *p* = 0.003	22.50	6.16	*t*(7) = −0.80, *p* = 0.447

Regarding reading and writing skills, SJ showed low performances on all tests except for the hiragana character reading test and the hiragana and katakana character writing tests, compared to Japanese monolinguals. In Japanese kanji, his reading and writing age was estimated as that of 7-year-olds. His reading and writing test performances were all significantly lower than bilinguals (see Tables 6, 9). Notably, his performance on the katakana word writing test differed from only bilingual girls (Table 9).

**Figure 1:**
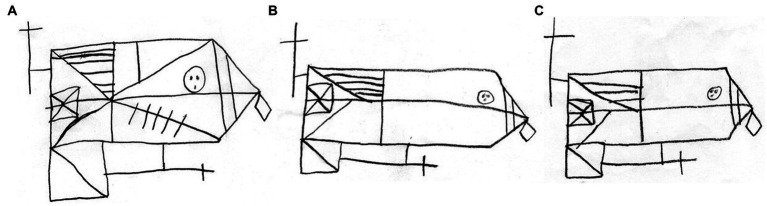
SJ’s drawings on Rey–Osterrieth Complex Figure Test (ROCFT). Figures **A**, **B**, and **C** depicts SJ’s copy, immediate recall, and delayed recall drawings, respectively.

Out of a series of receptive vocabulary tests, SJ showed low performance on only the shortened version of SCTAW, compared to both monolinguals and bilinguals. Regarding phonological skills, SJ’s performance on the non-word repetition test was lower than that of monolinguals but not bilinguals. In addition, his performance on the word backward span test was lower than those of both monolinguals and bilinguals, in terms of only RTs. Similarly, SJ’s performance on the RAN test was lower than those of both monolinguals and bilinguals. Regarding visual skills, SJ showed lower performance only on the copy drawing of the ROCFT compared to both monolinguals and the mixed gender of bilinguals (Table 7). Notably, SJ’s immediate and delayed recall scores on the ROCFT significantly differed from bilingual boys but not bilingual girls (Table 9). Figure 1 depicts SJ’s drawings on the ROCFT.

## Discussion

Table 10 summarizes the results of the assessments in Korean and Japanese.

**Table 10:** Summary of SJ’s low abilities based on the results of the assessments.

	SJ vs. monolinguals	SJ vs. bilinguals
**Reading accuracy**		
Korean	✓	
Japanese kana	✓	No data
Japanese kanji	✓	✓
**Spelling/writing accuracy**		
Korean		
Japanese kana	✓	✓
Japanese kanji	✓	✓
**Reading fluency**		
Korean	✓	✓
Japanese	✓	✓
**Receptive vocabulary**		
Korean		
Japanese	✓ (for only the shortened SCTAW)	✓
**Phonological skills**		
Korean	✓	✓
Japanese	✓	✓
**Naming speed**		
Korean	✓	✓
Japanese	✓	✓
**Visual skills**	✓	✓

In the comparison of the norm of monolingual children, a check mark (✓) indicates that SJ’s performance was below 1.5 SD from the mean of monolingual children (i.e., low performance). In the comparison of the norm of bilingual participants, a check mark (✓) indicates that a significant difference was obtained by the modified *t*-test.

Regarding SJ’s literacy profile, only his spelling accuracy in Korean was not lower than both monolinguals and bilinguals. In contrast, his reading accuracy was lower than monolinguals in both languages. SJ’s first language, Korean, is a heritage language in Japan. Hangul is common only among communities of Korean people or in Korean towns in Japan. In addition, SJ received Korean language classes at school only a few times per week. Therefore, SJ might have fewer less reading experiences in Korean than Korean monolingual children who live in Korea. Importantly, SJ and bilingual participants learned Korean from the same teachers using the same textbooks. This means that educational circumstances in learning Korean were similar between them. Since SJ’s reading accuracy in Korean did not differ significantly from that of bilinguals, fewer reading experiences might have resulted in his low reading accuracy relative to monolinguals. In contrast, Japanese is the main language in Japan. SJ used Japanese to learn subjects at school, as did Japanese monolingual children and bilingual participants. In contrast to Korean, SJ’s reading and writing accuracy in Japanese was lower than even bilingual participants. Therefore, his low accuracy in reading and writing Japanese cannot be explained by bilingualism. Meanwhile, SJ’s reading fluency was lower than that of even the bilinguals, in both languages. Thus, there was a marked dissociation of SJ’s literacy skills between Korean and Japanese in terms of accuracy.

The dissociation between languages was also observed in receptive vocabulary. SJ’s receptive vocabulary in Korean was not low. In contrast, out of receptive vocabulary tests in Japanese, SJ showed low performance only on the shortened version of the SCTAW. In the definition of developmental dyslexia, Lyon et al. (2003) state: “[s]econdary consequences may include problems in reading comprehension and reduced experience that can impede the growth of vocabulary and background knowledge” (p. 2). SJ informed us that he did not like reading books in Japanese and seldom read them. Therefore, he may have lacked reading experience in Japanese relative to the bilingual participants. Consequently, his performance on the shortened version of SCTAW might have been lower than monolingual and bilingual children. However, SJ’s score on the full version of SCTAW was within the average range of Japanese monolingual children. Therefore, SJ’s receptive vocabulary in Japanese appeared to be slightly low, but not delayed.

Notably, SJ did not show the dissociation of a cognitive profile between languages. His phonological skills and naming speed were impaired in both languages. In a series of visual skills tests, SJ showed low performances only on the ROCFT. His copy drawing lacked some components, some were added, and some were placed in the wrong position. These errors were not explained by inattention defined as symptoms of ADHD and clumsiness (see section Case history of SJ).

Why did SJ show dissociation of literacy skills between Korean and Japanese in terms of accuracy, despite that his cognitive profiles were common between the languages? As described in the Introduction section, the dissociation of literacy skills can result from differences in orthographic characteristics and cognitive demands on the development of literacy skills between languages. We discuss possible accounts for his literacy profile from orthographic and cognitive perspectives.

### Accounts for SJ’s literacy profile from the orthographic perspective

Based on the hypothesis of granularity and transparency (Wydell and Butterworth, 1999), we hypothesized that a Korean–Japanese bilingual child will be unlikely to show developmental dyslexia in both Korean and Japanese. This is because, in Hangul and Japanese kana, the correspondence between letters/characters and sounds is transparent, while, in Japanese kanji, the phonological unit in sounds corresponding to characters is large (see Table 1). Contrary to this prediction from the orthographic perspective, SJ’s reading skills in Japanese were impaired in terms of accuracy.

However, a ceiling effect in the norms of Japanese monolinguals can account for low reading accuracy in Japanese kana. In all reading accuracy tests in Japanese kana, the ceiling effect was observed in the norms of Japanese monolinguals (see Table 6). It does not necessarily mean that the tests are too easy. The ceiling effect reflects the fact that with Japanese kana (transparent orthography), it is easy to acquire character-to-sound correspondences (Wydell and Butterworth, 1999). SJ showed a perfect score or made one or two errors in each reading test in Japanese kana. Considering only a few reading errors per test, the ceiling effect in the norms of monolinguals might have resulted in SJ’s low performance on the reading tests in Japanese kana.

In contrast, in the 126 kanji word reading test of the STRAW-R, the norm of Japanese monolinguals did not show a ceiling effect. Moreover, SJ’s score on the test was significantly lower than that of the bilingual participants. This means that a ceiling effect and bilingualism are ruled out as reasons for his low reading accuracy in Japanese kanji. Therefore, we concluded that SJ had an impairment in reading accuracy in Japanese kanji. His reading deficits in Japanese kanji were inconsistent with the expectation from the orthographic perspective based on the hypothesis of granularity and transparency (Wydell and Butterworth, 1999).

### Accounts for SJ’s literacy profile from the cognitive perspective

As Table 1 shows, visual skills predict the reading and writing accuracy of monolingual children in Japanese (for kana, Uno et al., 2007; Inomata et al., 2016; Hamada and Uno, 2021; for kanji, Koyama et al., 2008) but not in Korean (e.g., Park and Uno, 2012, 2015; Ju et al., 2016). From a cognitive perspective, we hypothesized the following. If a Korean–Japanese bilingual child has deficits in visual skills, the child’s reading and writing difficulties in Japanese will be more remarkable than those in Korean. SJ’s reading and spelling accuracy in Korean did not differ from bilinguals significantly. In contrast, his reading and writing accuracy in Japanese was lower than even bilinguals. In addition, his visual skills were poor. Thus, SJ’s cognitive and literacy profiles matched this prediction.

Moreover, visual skills are more important in learning Japanese kanji than kana (Koyama et al., 2008). Therefore, we hypothesized that low visual skills would result in severe deficits in the reading and writing of Japanese kanji, relative to kana. In the hiragana, katakana, and kanji word reading and writing tests for sixth graders in the STRAW-R, the same words were used as stimuli (e.g., the word /tane/, seed in English, was used in each test as the hiragana stimulus “たね,” the katakana stimulus “タネ,” and the kanji stimulus “種”). As depicted in Table 6, SJ’s reading and writing scores in kanji were lower than those in hiragana and katakana. Thus, SJ’s accuracy in reading and writing Japanese matched this prediction as well.

In contrast to reading accuracy, reading fluency of Korean and Japanese monolingual children is predicted by phonological skills and naming speed (see Table 1). This means that common cognitive abilities involve in the development of reading fluency in the languages. Therefore, we hypothesized that a Korean–Japanese bilingual child might not show the dissociation of reading fluency between languages. SJ’s profile matched the prediction, again. In other words, he showed low reading fluency in both languages, along with deficits in the two cognitive abilities in each language.

In summary, SJ’s literacy profile matched the predictions from the cognitive perspective. Thus, our case study supports that the literacy profile of a bilingual child depends on the cognitive demands of the development of literacy skills in each language. To our knowledge, this study is the first to report that low visual skills can cause the dissociation of literacy profiles between languages. Previous studies have shown that the reading development of bilingual or biliterate children is predicted by their phonological abilities and naming speed (e.g., Lafrance and Gottardo, 2005; Farran et al., 2012; Bellocchi et al., 2017), in addition to orthographic characteristics. As new findings, this study suggests that visual skills also involve in the development of literacy skills in bilingual or biliterate children. The literacy development in some languages (like Japanese) is affected by visual skills, while it is not so in other languages (like Korean). Therefore, this case report implies that a cross-linguistic or bilingual study should consider differences in the relationship between visual skills and the development of literacy skills, between languages. Considering that some children with developmental dyslexia even in alphabetic languages possess weaknesses in visual skills (Huestegge et al., 2014), we should assess the visual skills of a bilingual child in addition to phonological skills and naming speed.

## Limitations

This is a case study. Therefore, it is impossible to generalize our findings. We believe that the comparison of SJ’s performances with those of his bilingual peers provided useful information on whether SJ’s low performance was due to bilingualism. However, the sample size of the Korean–Japanese bilingual participants was limited. In future studies, more bilingual participants are required. We noted that it is difficult to find many Korean–Japanese bilingual and biliterate children who live in Japan. This is because Korean is a minor language in Japan, and generally, primary schools have no Korean language classes.

We used the data on only bilingual participants whose scores on the RCPM were within the average range. SJ’s score on the RCPM was also within the average range. Thus, we could match at least non-verbal intelligence between SJ and the bilingual participants. However, SJ’s Full Scale IQ (FSIQ) of WISC-IV was below 1 SD from the average. We did not administer the WISC-IV to the bilingual participants. Therefore, the FSIQ of each bilingual participant was not known. This is one of the limitations of this study. However, SJ’s performances were not necessarily low on all the tests used in the Korean and Japanese assessments. Therefore, the influence of this issue might have been limited. To clarify this, future studies should match the FSIQ between a target case and bilingual controls.

In addition, SJ’s working memory was low based on the working memory index (WMI) of the WISC-IV. It might be possible that his low working memory could affect his slow reaction times on reading and cognitive tests. However, it is unclear how working memory is associated with naming speed and reading fluency in Korean and Japanese. We did not measure the working memories of bilingual participants in this study. Future studies should investigate the relationship between working memory and reaction times in Korean and Japanese cognitive and reading tests.

## Conclusion

We reported on a Korean–Japanese bilingual boy with developmental dyslexia (aged 11), SJ. He showed some differences in the profile of literacy skills between Korean and Japanese, although his cognitive profile was similar between languages. Particularly, in terms of accuracy, his reading and writing skills were dissociated between Korean and Japanese. Moreover, his low reading and writing accuracy was more remarkable in Japanese kanji. The dissociation was explained by the fact that visual skills influence the reading and writing accuracy of monolinguals in Japanese, but not Korean. Our case, SJ, supports the cognitive perspective, namely, the idea that cross-linguistic differences in cognitive demands on the development of literacy skills can cause dissociation in the literacy profile of a bilingual child between languages.

## Data availability statement

The raw data supporting the conclusions of this article will be made available by the authors, without undue reservation.

## Ethics statement

This study was conducted after obtaining approval from the Ethics Committee of Osaka Kyoiku University, in accordance with the Declaration of Helsinki. The school principal of the participants and SJ’s parents provided written informed consent. Participants (including SJ) provided oral informed consent, which was recorded using an IC recorder.

## Author contributions

AS was mainly responsible for data collection of assessments in Japanese and analysis and completed the draft. YJ was involved in data collection of assessments in Korean and the analysis and writing of parts of the manuscript. AU revised the manuscript. All authors contributed to the article and approved the submitted version.

## Funding

This research was supported by JSPS KAKENHI (grant numbers 15K17420, 19K14297, and 22K13746).

## Conflict of interest

The authors declare that the research was conducted in the absence of any commercial or financial relationships that could be construed as a potential conflict of interest.

## Publisher’s note

All claims expressed in this article are solely those of the authors and do not necessarily represent those of their affiliated organizations, or those of the publisher, the editors and the reviewers. Any product that may be evaluated in this article, or claim that may be made by its manufacturer, is not guaranteed or endorsed by the publisher.
